# Early Initiation of a Standardized Open Abdomen Treatment With Vacuum Assisted Mesh-Mediated Fascial Traction Achieves Best Results

**DOI:** 10.3389/fsurg.2020.606539

**Published:** 2021-02-09

**Authors:** Frederik Berrevoet, Silvio Lampaert, Kashika Singh, Kamilya Jakipbayeva, Stijn van Cleven, Aude Vanlander

**Affiliations:** Department of General and Hepatopancreaticobiliary Surgery and Liver Transplantation, Ghent University Hospital, Ghent, Belgium

**Keywords:** open abdomen, dynamic closure, negative pressure therapy, fascial closure, abdominal compartment syndrome, mesh mediated fascial traction

## Abstract

**Background:** The open abdomen (OA) is an important approach for managing intra-abdominal catastrophes and continues to be the standard of care. Complete fascial closure is an essential treatment objective and can be achieved by the use of different dynamic closure techniques. Both surgical technique and—decision making are essential for optimal patient outcome in terms of fascial closure. The aim of this study was to analyse patients' outcome after the use of mesh-mediated fascial traction (MMFT) associated with negative pressure wound therapy (NPWT) and identify important factors that negatively influenced final fascial closure.

**Methods:** A single center ambispective analysis was performed including all patients treated for an open abdomen in a tertiary referral center from 3/2011 till 2/2020. All patients with a minimum survival >24 h after initiation of treatment were analyzed. The data concerning patient management was collected and entered into the Open Abdomen Route of the European Hernia Society (EHS). Patient basic characteristics considering OA indication, primary fascial closure, as well as important features in surgical technique including time after index procedure to start mesh mediated fascial traction, surgical closure techniques and patients' long-term outcomes were analyzed.

**Results:** Data were obtained from 152 patients who underwent open abdomen therapy (OAT) in a single center study. Indications for OAT as per-protocol analysis were sepsis (33.3%), abdominal compartment syndrome (31.6%), followed by peritonitis (24.2%), abdominal trauma (8.3%) and burst abdomen (2.4%). Overall fascial closure rate was 80% as in the per-protocol analysis. When patients that started OA management with MMFT and NPWT from the initial surgery a significantly better fascial closure rate was achieved compared to patients that started 3 or more days later (*p* < 0.001). An incisional hernia developed in 35.8% of patients alive with a median follow-up of 49 months (range 6–96 months).

**Conclusion:** Our main findings emphasize the importance of a standardized treatment plan, initiated early on during management of the OA. The use of vacuum assisted closure in combination with MMFT showed high rates of fascial closure. Absence of initial intraperitoneal NPWT as well as delayed start of MMFT were risk factors for non-fascial closure. Initiation of OA with VACM should not be unnecessary delayed.

## Introduction

Open abdomen (OA) is a well-known clinical entity. It leaves a laparotomy incision without closure and is to be distinguished from “burst abdomen”, which is an unintended fascial dehiscence after primary closure of a laparotomy incision. Its objective is to temporarily close the abdomen in a tension-free manner and to allow second-look operations. This surgical strategy is now used for managing different pathologies, e.g., intra-abdominal hypertension, sepsis, trauma or staged abdominal wall repair (1). Although this procedure is potentially life-saving, it is also associated with a number of complications and with a high mortality (2, 3). In order to reduce both the complications associated with open abdomen and to improve fascial closure rates, the preferred method of approach now focusses on early closure of the abdomen, preferably within the first 10–14 days (4). There have been several ways of temporary abdominal wall closure (TAC) which help closing the fascia. However, little is known about reasons for non-fascial closure at the end of open abdomen treatment (1, 5). Early planning and an upfront surgical strategy are key-elements. In relation to the overall outcome of an open abdomen treatment, the classification scheme reported and amended by Björk et al. correlates with prognosis and is very helpful in determining both fascial closure rate as well as overall morbidity and mortality (6, 7). An important distinction should be made between the so-called static and dynamic closing techniques. The combination of negative-pressure wound therapy (NPWT) and mesh-mediated fascial traction (MMFT) or NPWT and dynamic fascial sutures (DFS) is associated with highest fascial closure rates (8–10). The purpose is to establish edema reduction in combination with fascial reapproximation (11–13).

Currently, vacuum assisted closure (VAC) in combination with MMFT (VACM) represents the current gold standard with fascial closure rates of up to 90% and is acknowledged to be superior to other techniques lacking mechanical fascial traction (14–17). Recently, the European Hernia Society (EHS) published clinical guidelines on the management of the open abdomen and clearly recommended dynamic closure techniques, with 75.9 vs. 33.9% fascial closure rate compared to the results of static closure techniques (18). The aim of this analysis is to evaluate patient outcome after VACM and to determine crucial factors for optimal treatment, regarding both timing and surgical technique.

## Materials and Methods

### Study Population and Study Design

From 3/2011 till 2/2020, all patients treated with intraperitoneal NPWT at our tertiary referral hospital were both retrospectively and prospectively entered into the Open Abdomen Route of the European Registry of Abdominal Wall Hernias (EuraHS—www.eurahs.eu) (19, 20). As the Open abdomen Route only became available in 2015 all data that was already gathered before was retrospectively entered in EuraHS. Approval of the Medical Ethics Committee was obtained prior to this study.

All files of patients whom underwent VACM at our hospital in this period were retrospectively analyzed. Patients with NPWT without MMFT and patients with only use of MMFT were excluded. Patients who died within 24 h after initiation of open abdomen treatment were also excluded. Variables on every patient and course of treatment were registered including underlying conditions and comorbidities, open abdomen management, clinical course, and clinical follow-up assessments.

### VACM Protocol

A standardized protocol was used in all cases as previously described by Petersson et al. (21). At time of initial surgery an intraperitoneal NPWT device was placed when no new anastomosis, bile leak or active bleeding was present. This abdominal dressing (ABThera™ Open Abdomen Negative Pressure Therapy System, KCI, San Antonio, TX) consists of an elliptical shaped perforated polyurethane foam encapsulated in a visceral protective layer, designed to be wrapped around the viscera. It's mandatory for the device to be placed deep in the paracolic gutters and Douglas space, in order to evacuate as much liquid as possible and to avoid formation of adhesions.

**Figure 1:**
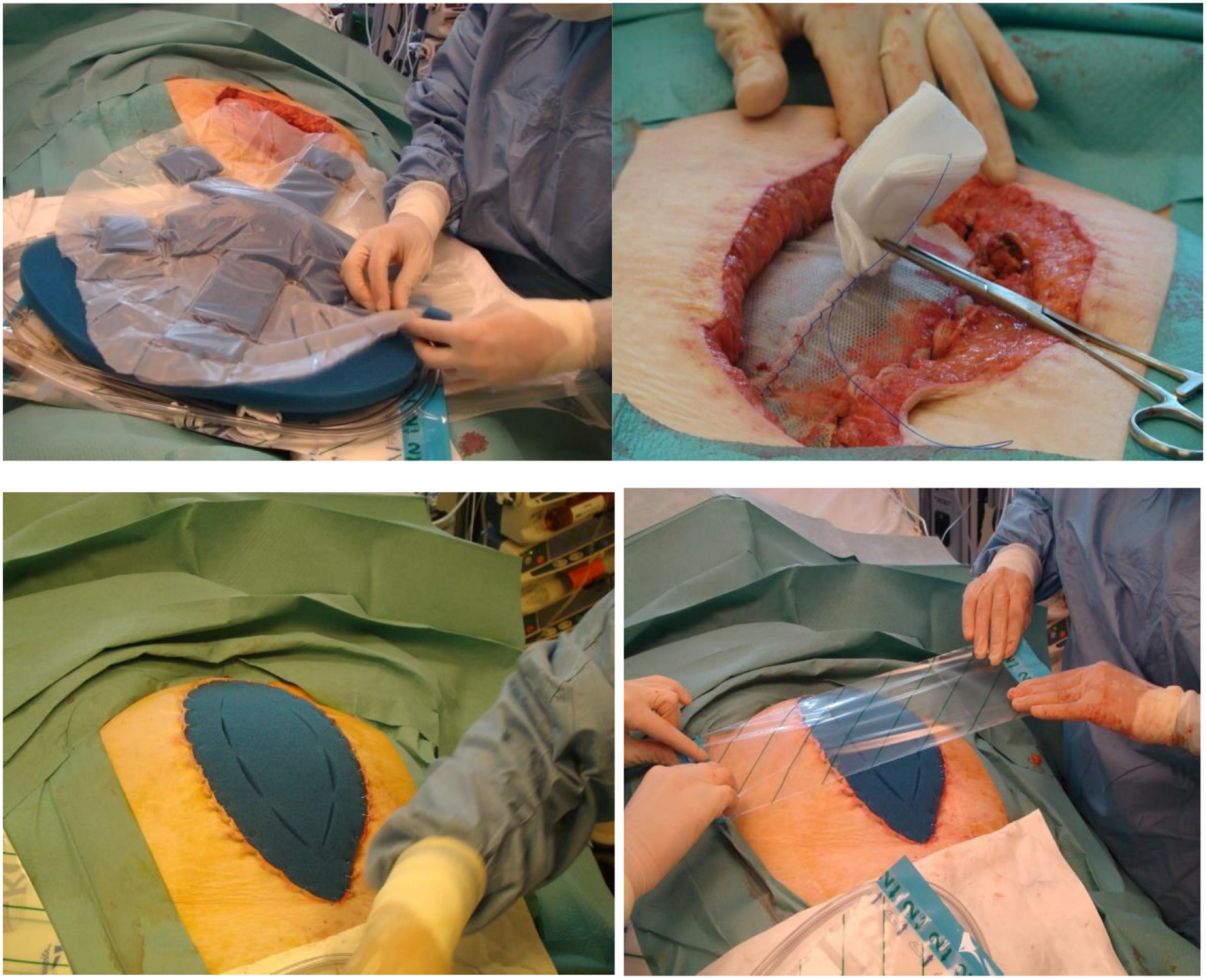
Technique of vacuum assisted mesh mediated fascial traction.

In other cases a plain plastic sheet was used as a visceral protective layer. On top of the visceral protective layer a heavyweight mesh is sewn in with a continuous non-resorbable monofilament 2/0 suture at the fascial edges. Strong traction on this mesh is then applied. The mesh is then covered by a macroporous oval shaped foam dressing and protected by an adhesive sheet with attachment of the suction pad and connected to a canister. Suction was applied at −125 mm Hg (Figure 1).

### Outcome Variables

Our primary outcome was delayed primary fascial closure, i.e., the fascial edges completely sutured together with no remaining fascial defect.

### Patient Characteristics

The Open Abdomen Route covers many variables on every patient and course of treatment and is divided into various categories providing information on the patient, underlying conditions and comorbidities, open abdomen management, clinical course, and clinical follow-up assessments.

Sex, Body Mass Index (BMI), age at time of surgery, indication for OA therapy, time between initial surgery and start of VACM, duration of VACM, complications and patient mortality were variables chosen for analysis based on their clinical relevance in regard to open abdomen management. Classification of the open abdomen was based on Björck's classification published in 2009 (7).

Follow-up was performed by chart-review at the time of analysis and in case patients were still alive a clinical examination was performed to evaluate incisional hernia formation.

### Statistical Analysis

A descriptive analysis was performed on the different targets of the VACM protocol and patient-related factors. Statistical analysis was performed using SPSS (version 25.0) software. Normally distributed variables were presented as means ± standard deviations. Non-normally distributed variables were presented as medians and 95% confidence intervals (CIs). Depending on the distribution and the level of measurement, univariate analyses were performed using Fisher's exact test, chi-squared test or Mann-Whitney U test. The significance threshold was set at *p* = 0.05.

## Results

### Patient Characteristics of the Complete Study Cohort

Between 08/03/2011 and 20/02/2020 152 patients underwent an open abdomen treatment using VACM. Thirty-two patients were excluded for final analysis of the primary endpoint because they died before final closure of the abdomen or within 24 h after closing the abdomen (9).

The mean age of the patients was 58 years. Sixty-eight percent were male. The mean BMI was 26.0 at the initiation of open abdomen management. Overall hospital mortality was 21% (32 of 152 patients). Baseline characteristics and risk factors regarding abdominal wall closure and wound healing are depicted in Table 1.

**Table 1:** Patients characteristics.

Number of patients	152
Age (years)	57.53 ± 16.3
Gender (female/male)	82 (68.3%)/38 (31.7%)
Body mass index (BMI)	26.15 ± 5.9
Malignancy	18 (15%)
Diabetes	19 (15.8%)
Cardiopulmonary disease	32 (26.7%)
Immunosuppression	7 (5.8%)
Mannheim Peritonitis Index (MPI)	20 ± 6
Injury Severity Score (ISS)	23 ± 20
In-hospital mortality	32/152 (21.1%)
Type of per protocol incision (midline/transverse/combined, *n =* 120)	99 (82.5%)/16 (13.3%)/5 (4.1%)
Björck's classification at the initiation of OAT	Grade 1A–clean OA (65.8%)
	Grade 1B–contaminated OA (34.2%)
	Grade 2A–clean OA developing adherence (0%)
	Grade 2B–contaminated OA developing adherence (0%)
	Grade 3–OA complicated by fistula (0%)
	Grade 4–frozen OA (0%)
Björck's classification at the completion of OAT	Grade 1A–clean OA (83.7%)
	Grade 1B–contaminated OA (16.3%)
	Grade 2A–clean OA with adherence (0%)
	Grade 2B–contaminated OA with adherence (0%)
	Grade 3–fistula (0%)
	Grade 4–frozen abdomen (0%)

### Patients Completing the Open Abdomen Treatment

Indications were noted as sepsis in 40 patients (33.3%), abdominal compartment syndrome in 38 (31.6%), peritonitis in 29 (24.2%), trauma in 10 (8.3%), and burst abdomen in three patients (2.5%).

In the per-protocol analysis a midline incision was used in 99 patients (82.5%). In 16 patients there was a transverse incision (13.3%) and in 5 a combined incision (4.1%) was used.

**Figure 2:**
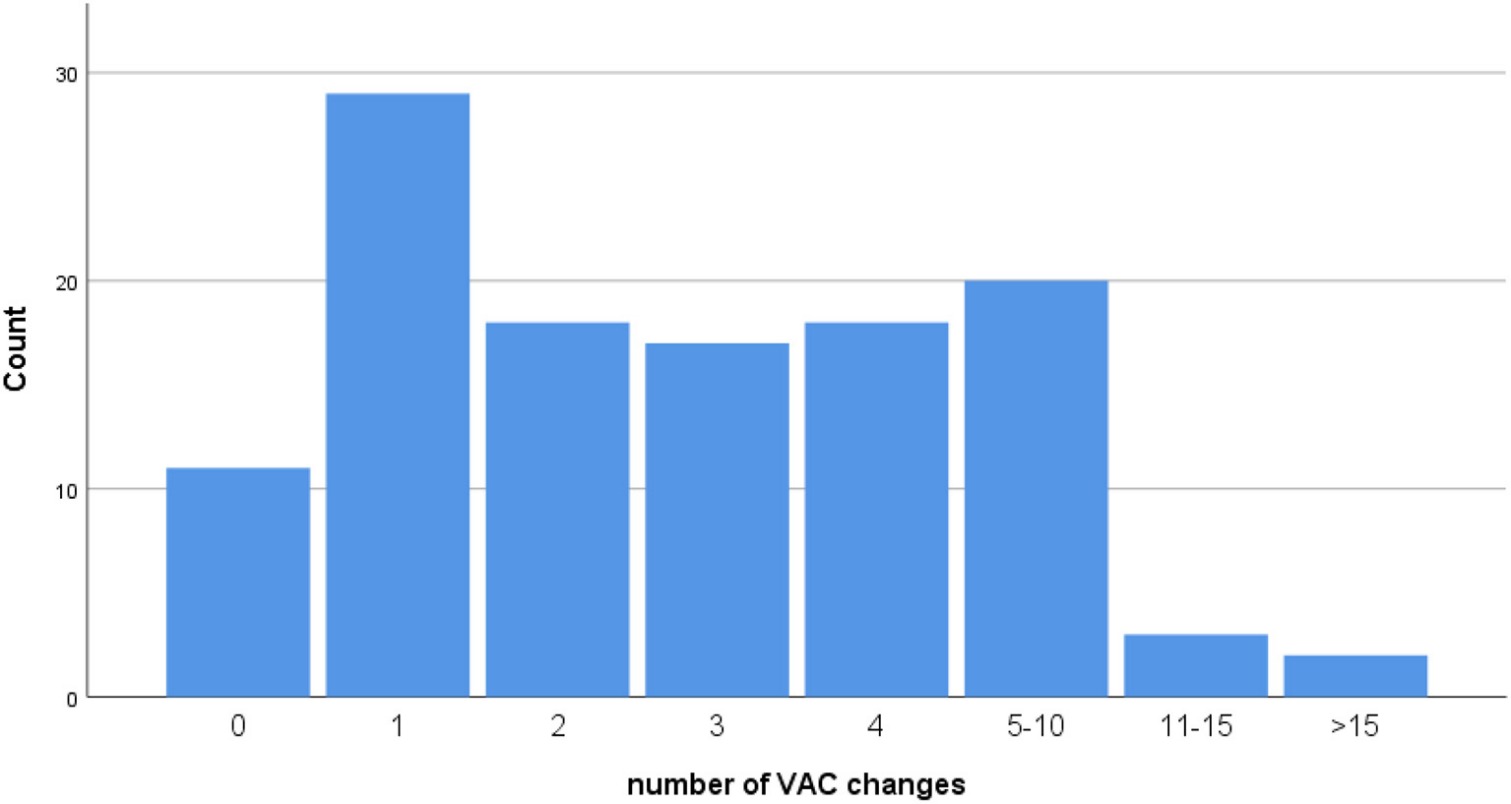
Number of VAC changes classified according to EuraHS.

The average duration of the OAT was 13 days (range 1–93 days). The abdomen of 11 patients (9%) was closed within the next operation. Most patients (24%) needed 1 change of the temporary closure. It was evenly distributed for 2, 3, 4, and 5–10 changes of the closure, namely 15%. Only four patients needed more than 11 operations to close the abdomen (Figure 2). Considering the different indications for OA therapy trauma patients had the shortest mean closure time (2.6 days), 7.2 days for burst abdomen, 12.4 days for peritonitis patients, and 15.1 days for the patients with sepsis.

The overall fascial closure rate in the per-protocol analysis after VACM in our study population, being our primary endpoint, was 80% (96 of 120 patients), which excluded patients who had died during OA treatment. In the intention-to-treat analysis (including patients that died during treatment) the fascial closure rate was 63.2% (96 of 152 patients). An anatomical closure (fascial closure + subcutaneous and skin closure) was immediately performed in 90 patients (75%). In 6 (5%) the fascia was sutured, but the superficial layers were closed using NPWT. Only one patient needed a bilateral component separation to close the anterior fascia.

The group of patients that needed a short period of OA management and only 1-2 NPWT changes reached fascial closure in 80% of cases, while the groups that needed 7–21 days of OA management did show a closure rate of 78% (Table 2).

**Table 2:** Treatment characteristics.

	**Number of patients**	**%**	***p*-value**
Complete fascial closure (per-protocol analysis)	96/120	80	
Complete fascial closure (intention-to-treat analysis)	96/152	63.2	
Fascial closure rates according to OA indication			0.01
Trauma	9/10	90.0	
Peritonitis	24/29	82.8	
Abdominal compartment syndrome	31/38	81.6	
Burst abdomen	1/3	33.3	
Sepsis	31/40	77.5	
Duration of OAT		Fascial closure (%)	0.62
<7 days (1–2 reoperations)	50	80	
7–21 days (3–6 reoperations)	47	78	
>21 days (7 or more reoperations)	23	78	

Considering the classification of OA according to Björk, most patients in our series had a Grade 1A or 1B. We did not observe any patients with frozen abdomen as all patients had their initial surgery as well as the decision for OA treatment in our hospital. During every change of the NPWT, both the abdomen and the viscera are flushed and gently mobilized, especially at the level of the abdominal wall. As this happens 2× a week, frozen abdomen is not an issue. If final fascial closure is not feasible, at the last change and closure of the abdomen, we replaced the non-absorbable mesh by a absorbable mesh and skin closure. This did not cause any fistulae.

### Analysis of Non-fascial Closure

When analyzing the determining factors for non-closure of the fascia, 19 out of 24 patients that could not be closed, started their initial VACM 3 or more days after the index procedure with leaving the abdomen open (79.2%). Reasons for not starting VACM at initial surgery were risk for postoperative bleeding (*n* = 14), fear for anastomotic leakage *n* = 4 and risk for biliary fistula (*n* = 1). In those cases a type of Bogota bag was installed without NPWT nor mesh placement. Only four out of 24 got their fascia edges closed despite starting late (20.8%). Out of the patients that were not closable after OA management, there was a significant difference between patients started their VACM immediately at the time of initial surgery (5/96, 5.2%) vs. patients with a late start of their VACM (19/24, 79.2%; *p* < 0001). Mortality 12 months after closure was 4.2% (1/24 patients) vs. 3.1% (3/96 patients; *p* = 1.0). Median length of hospital stay of the analyzed 120 patients was 54 days (range: 4–275 days).

### Development of Incisional Hernia During Follow Up

Considering the follow-up of this specific cohort of patients, an incisional hernia developed in 35.8% of patients considering the per-protocol analysis; all patients in which fascial closure could not be achieved (*n* = 24) developed an incisional hernia, of which only seven had a mesh repair. The other 17 did not want their hernia defect repaired (70.8%). Out of the 90 patients with fascial closure, 19 developed an incisional hernia as well (21.1%), and 15 had an abdominal wall repair with retromuscular mesh (78.9%). The median follow-up period of 49 months (range 6–96 months).

## Discussion

The present study analyzed a large patient cohort with OA for several indications. When treating this type of patients with necessity for an open abdomen management, time strategy is of utmost importance, as closure of the abdominal wall, i.e., fascial closure, should be aimed within 10–14 days. Initial therapy during the first 24–48 h should not only be focused on adequate edema- and excessive fluid removal as well as on hemodynamic stabilization. Surgeons should be thinking about how to handle the abdominal wall, to evaluate its compliance and finally how to obtain fascial closure. It is well-know that a non-closed abdominal cavity poses an increased risk for complications, not in the least entero-atmospheric fistulae. Closure is mandatory at the earliest possibility (22).

The EHS clinical expertise guidelines strongly recommended the use of dynamic closure techniques over other (static) techniques to achieve best fascial closure rates and low morbidity and mortality (18).

Our main findings in this analysis emphasize the importance of a structured treatment plan, initiated early on during management of the OA since the use of VACM showed high rates of fascial closure. The absence of initial intra-abdominal NPWT as well as a delayed start of MMFT, or the combination of both, were associated with a high risk of non-fascial closure. Cirocchi et al. also found better outcomes with NPWT when compared to techniques without NPWT (1). The difference in fascial closure rates was not significant and emphasizes the fact that NPWT alone may not be able to sufficiently prevent fascial lateralization during OA treatment (23). In some reports we see fascial closure rates drop to 60% or even to 30% in postponed NPWT/MMFT (24, 25). A recent review specifically focused on dynamic closure techniques only. The combination of NPWT and progressive fascial traction to the midline gives an overall closure rate between 72 and 93% (26). Main reasons for not to immediately initiate VACM in our study were bleeding/oozing at first laparotomy, bile leakage after severe liver trauma or a concomitant bowel anastomosis during the initial surgery. Traction was also not always applied from the start of OA as for some patients quick closure was initially expected. As these patients had significantly less fascial closure achieved than patients with immediate start of MMFT and NPWT, a clear message would be to better use a mesh too many than diminishing the chances for complete fascial closure. Surgeon's experience does not play an important role in this decision making, as both the initial surgery and the decision for OA management were always performed by a senior surgeon, familiar with the mesh mediated fascial traction technique.

Another point of attention using the MMFT technique is the use of a permanent heavy weight, small pore mesh for traction. We believe this is essential in these indications in which heavy traction should be applied on the fascial edges. Large pore meshes are not suitable for this purpose as they are too elastic and will be torn during the process.

The distribution of our patient population and the indications for OA management reflect those commonly found in the literature: the most frequently reported indications for OA were peritonitis or sepsis, followed by ACS, and trauma. The differences in mortality rates most likely reflect differences in patient population and only to a lesser extent imply a direct effect of the applied dynamic closure technique. In our study, in-hospital mortality of 21% was in line with the literature for OA, which varies between 10 and 45% (16, 27, 28).

There was a difference in fascial closure rates between the various indications for OA treatment in our series and the highest rates were observed for trauma patients (90%), which can be explained by a combination of the need for a short treatment period and less systemically ill patients, as shown by Montori et al. (29). In case fascial closure might take longer, there has been published sparse data on the use of Botulinum Toxin A (BTA) in OA management by Zielinski and colleagues in 18 patients (30). This toxin functions by blocking the release of acetylcholine and pain modulators (calcitonin gene–related peptide and substance P) from the pre-synaptic cholinergic nerve terminal, resulting in flaccid paralysis and pain modulation. If this paralysis may diminish lower midline abdominal wall tension, the rate of primary fascial closure might increase. However, at the time of life-saving surgical procedures or trauma, it is neither indicated nor possible to obtain informed consent from patients. Alternatively, the procedure for injection of BTA can be performed during a return trip to the OR. The clinical effect of this paralysis can be demonstrated as early as day 3 after intramuscular injection with maximum effect reached at 2 weeks (31). In the series of Zielinski et al. the primary fascial closure rate was 83% with a partial fascial closure rate of 6% and a planned ventral hernia rate of 11%, but no comparative analysis was performed with patients without BTA injections. There were no complications related to BTX (29). Surprisingly, no other reports have been published using this approach since.

Despite all efforts to finally obtain full fascial closure in OA patients, the longterm follow-up of these patients in terms of incisional hernia rate is scarce, and rather worrisome (21, 32, 33). The incidence of incisional hernias ranged from 21% at 21 months to 54% after 5 years of follow-up. The repair rate in these series differed and was 33 and 42%, respectively. In our series the incisional hernia rate was, as can be expected, 100% for patients in which fascial closure could not be obtained, but it is rather remarkable that only seven out of these 24 patients requested a hernia repair (29.2%).

Bjarnason and co-workers reported their 1-year follow-up after MMFT in combination with NPWT and described 66% of incisional hernias in these patients using CT evaluation (34). Despite the fact that more patients can be closed after OAT using fascial traction in combination with NPWT, the focus for these patients should now more and more be on how to prevent incisional hernias developing after final fascial closure in this severely ill patient population. Petersson et al. recently published a small series in which an onlay mesh was applied early during treatment by suturing to the fascia in two rows with a 3- to 4-cm overlap from the midline incision, used for traction and kept for reinforced permanent closure. A total of 11 patients were treated with a fascial closure rate of 100% and a 30 days mortality of 0%. Only two out of nine patients developed a hernia. Neither of the hernias were symptomatic nor clinically detectable. Therefore, this reinforced fascial closure might help toward a decreased long-term incisional hernia rate (35).

Our study has several limitations: in the absence of sufficiently large numbers of patients, a multivariate analysis has not been performed to assess the effects of different factors on fascial closure rates. Secondly, despite the fact that it is an ambispective dataset, this single center analysis involves OA patients with different etiologies. This leads to a heterogeneous mixture of parameters and without multivariate analysis the influence on fascial closure rate is difficult to estimate.

In conclusion, the analysis of this large cohort of open abdomen patients confirmed that VACM is an effective and safe technique and achieves good results regarding delayed fascial closure. It is important to realize that several factors are key in achieving best outcomes and are related to early surgical decision making: 1. Fast start of intra-abdominal NPWT and 2. implementing fascial traction as soon as possible.

As comparative data considering the different techniques of dynamic closure are still lacking, NPWT should be used in combination with dynamic closure techniques and devices to obtain better insight in how to best treat these cohorts of patients in the future.

## Data Availability Statement

The raw data supporting the conclusions of this article will be made available by the authors, without undue reservation.

## Ethics Statement

The studies involving human participants were reviewed and approved by Ethical Committee of Ghent University Hospital. Written informed consent for participation were not required for this study in accordance with the national legislation and the institutional requirements.

## Author Contributions

FB was involved in design of the study, data analysis, and writing of the manuscript. AV was involved in design of the study and critical review of the manuscript. SC was involved in critical review of the manuscript. KJ and KS was involved in data gathering and critical review of the manuscript. SL was involved in data gathering, data analysis, and critical review of the manuscript. All authors contributed to the article and approved the submitted version.

## Conflict of Interest

The authors declare that the research was conducted in the absence of any commercial or financial relationships that could be construed as a potential conflict of interest.
